# The Impact of Pre-transplant Cell-free DNA Levels on Leukemia Relapse and Transplant-related Complications in Allogeneic Hematopoietic Stem Cell Transplant Recipients

**DOI:** 10.4274/balkanmedj.galenos.2020.2019.8.25

**Published:** 2020-04-10

**Authors:** Zeynep Arzu Yegin, Ferda Can, Sanem Gökçen, Rezzan Eren Sadioğlu, Zübeyde Nur Özkurt, Çiğdem İlhan, Münci Yağcı

**Affiliations:** 1 Department of Hematology, Gazi University School of Medicine, Ankara, Turkey; 2 Department of Internal Medicine, Gazi University School of Medicine, Ankara, Turkey

**Keywords:** Acute lymphoblastic leukemia, acute myeloid leukemia, allogeneic hematopoietic stem cell transplantation, cell-free DNA

## Abstract

**Background:**

Cell-free DNA, which may be considered as “liquid” biopsy, may serve as a diagnostic and prognostic marker not only in hematological malignancies but in solid tumors as well.

**Aims:**

To investigate the prognostic role of pre-transplant cell-free DNA levels in allogeneic hematopoietic stem cell transplant recipients.

**Study Design:**

Retrospective cohort study.

**Methods:**

A total of 177 allogeneic hematopoietic stem cell transplant recipients [median age: 36 (16-66) years; male/female: 111/66] with an initial diagnosis of acute leukemia were included in the study. Cell-free DNA was extracted from pre-transplant serum samples by using the MagNA Pure Compact Nucleic Acid Isolation Kit I with the MagNA Pure Compact instrument (Roche Diagnostics, Penzberg, Germany).

**Results:**

A positive correlation was demonstrated between cell-free DNA and age (p=0.018; r=0.177). Pre-transplant cell-free DNA levels were lower in bcr-abl (+) patients (p=0.001), while an adverse correlation was indicated between cell-free DNA and bcr-abl levels (p=0.001; r=−0.531). Acute lymphoblastic leukemia patients with bcr-abl positivity (p=0.001) or abnormal cytogenetics (p=0.038) represented significantly lower pre-transplant cell-free DNA levels. Cell-free DNA levels were lower in patients who developed sinusoidal obstruction syndrome (p=0.035). In terms of long-term complications, acute myeloid leukemia patients who experienced post-transplant relapse had significantly lower pre-transplant cell-free DNA levels (p=0.024). Overall survival was not statistically different between high- and low- cell-free DNA groups (45.2% vs 22.5; p=0.821).

**Conclusion:**

In general, low serum levels of pre-transplant cell-free DNA seem to be associated with transplant-related morbidities and may be considered an adverse prognostic factor for allogeneic hematopoietic stem cell transplant recipients.

Hematological malignancies may be considered as “liquid” tumors, because the primary involved sites are bone marrow and lymphoid tissues, along with neoplastic cells circulating in the peripheral blood (1). The amount of cell-free deoxyribonucleic acid (cfDNA) increased in patients with hematological malignancies besides a variety of solid tumors. Circulating cfDNA may sustain genetic characteristics of the tumor as cancer-specific mutations were demonstrated in the circulating cfDNA of acute myeloid leukemia (AML) and myelodysplastic syndrome (MDS) patients. In addition, tumor-associated micro-ribonucleic acids were identified in the peripheral blood of lymphoma patients (2,3). Circulating cfDNA, which may be described as “liquid” biopsy, is currently introduced as a diagnostic marker because it provides a typical representation of tumor genetic profile and has a feasible role in minimal residual disease (MRD) determination, which makes it a potential predictor of prognosis (2,4,5,6,7).

Cell-free DNA is composed of extracellular DNA fragments that are released from the cells as a result of necrosis or apoptosis. In healthy individuals, cfDNA is a product of physiological hematopoietic cell cycle, including myeloid and lymphoid apoptotic cells, whereas in cancer patients, only a small amount of total cfDNA originates from neoplastic cells. The wide range of this circulating tumor DNA fraction, which differs between 0.01% and 60% of total cfDNA, depends on several tumoral features, including volume, stage, vascularization, proliferation, and apoptosis rate (1,2,4,7,8,9).

To date, the predictor role of cfDNA was demonstrated in a wide spectrum of malignancies, including hematological disorders such as AML, MDS, and lymphoid neoplasms (2). In particular, this study was performed to evaluate the potential prognostic role of pre-transplant cfDNA levels in the development of transplant-related complications and survival in allogeneic hematopoietic stem cell transplantation (allo-HSCT) recipients.

## MATERIALS AND METHODS

The study cohort was composed of 177 acute leukemia patients who underwent allo-HSCT. Participants were classified into two subgroups for comparable statistical analysis, which were denominated as high- and low-cfDNA groups, based on the median cfDNA level, which was considered to be 8.5 (3.7–56.6) ng/μL. Patient demographics and transplant characteristics are presented in Tables 1 and 2.

Pre-transplant serum samples were stored at −80°C until the assay. Cell-free DNA was obtained from the serum on the basis of the manufacturer’s instructions by using MagNA Pure Compact Nucleic Acid Isolation Kit I and instrument (Roche Diagnostics, Penzberg, Germany). Four hundred microliters of serum specimen were used as the starting volume, and the nucleic acids were eluted in 100 μL of the supplied elution buffer. Cell-free DNA concentration, which is expressed in ng/μL, was measured at 260 nm and the purity was measured using the 260/280 ratio by using a Thermo Scientific™ *NanoDrop Lite* Spectrophotometer.

Median pre-transplant cfDNA level was 8.5 (3.7–56.6) ng/μL, whereas simultaneous measurement of white blood cell (WBC) count yielded a median of 4.6 (0.3–17.7)×109/L in the whole population. Given that WBC counts may interfere with cfDNA analysis, we performed a comparative analysis and did not find any significant statistical correlation between cfDNA levels and WBC counts. WBC counts were similar in high- and low-cfDNA groups. Therefore, additional processing such as logarithmic transformation was not required for statistical analysis.

### Statistical analysis

All 177 subjects had valid cfDNA data with a mean serum level of 9.576 ng/μL. On the basis of a standard deviation of 5.857, the standard error was estimated to be 0.440. The lower and upper boundaries of the 95% confidence interval (CI) were between 8.707 and 10.444 (p <0.001). The 95% CIs for cumulative survival were determined to be between -8.325 and 10.483 (p=0.821). The statistical power of the study was 95.23% on post hoc analysis.

Shapiro–Wilk test was performed for normality analysis. Parametric and non-parametric tests were used in case of normal and abnormal distribution, respectively. For normal distributed groups, equality and homogeneity of variances were analyzed by Levene’s test through one-way ANOVA and Student’s t-tests. Continuous variables were compared by conducting Student’s t-test for normal distribution, whereas Mann–Whitney U and Kruskal–Wallis tests were used for abnormal distributed parameters. Categorical variables were analyzed by chi-square test. Correlations were determined by Pearson and Spearman tests for normal and abnormal distributed data, respectively. Survival analysis was performed using Kaplan–Meier test. Cox regression and logrank tests were used for the assessment of risk factors that were significantly associated with survival. The statistical threshold for significance was considered to be p <0.05 on SPSS 22.0 (SPSS Inc, Chicago, IL, USA).

### Ethical standards

The study was certified by the institutional ethics board of the Gazi University Faculty of Medicine (date: 12.02.2018; number: 106). The procedures in the study were consistent with the Helsinki Declaration, and informed consent was obtained from all participants.

## RESULTS

Median cfDNA levels did not statistically differ in terms of gender; primary diagnosis; extramedullary leukemia; cytogenetics, including fms like tyrosine kinase 3 (FLT3), nucleophosmin (NPM-1), AML1-ETO (RUNX1/RUX1T1), inv16 (CBFB/MYH11), and complex karyotype; pre-transplant disease status, development of mucositis; peri-engraftment infections; cytomegalovirus (CMV) reactivation; thrombotic microangiopathy (TMA); graft versus host disease (GvHD); and relapse (p>0.05). Early and late transplant complications are detailed in Table 3.

Patients who presented bcr-abl positivity had significantly lower cfDNA levels than bcr-abl (-) patients [7.5 (5.4–12.1) vs 9.5 (7–13.4) ng/μL; p=0.001]. A negative correlation was identified between bcr-abl status and cfDNA amount (p=0.001; r=−0.531). Patients with sinusoidal obstruction syndrome (SOS) had lower levels of cfDNA compared with patients who did not develop SOS [7.7 (4.7–15.3) vs 8.8 (5.2–56.6) ng/μL; p=0.035]. In addition, a tendency to SOS was observed in patients who had lower pre-transplant cfDNA levels (p=0.049). An adverse relationship existed between cfDNA amount and SOS development (p=0.035; r=−0.163). Cytomegalovirus reactivation was also more frequent in patients with lower pre-transplant cfDNA levels, without statistical significance (p=0.065).

Peri-transplant infections were evaluated in 153 patients. Febrile neutropenia was demonstrated in 134 patients (87.6%). Thirty-seven patients (27.6%) had catheter-related infections, 19 patients (14.2%) had bloodstream infections, 15 patients (11.2%) had urinary tract infections, 11 patients (8.2%) had pneumonia, and 21 patients (15.7%) had other types of infections. In a total of 31 patients (23.1%), the site of infection or microbiological documentation was not identified. Seven patients developed invasive fungal infection. Any significant association was not indicated between cfDNA levels and infectious complications.

Median age was higher in the high-cfDNA group than in the low-cfDNA group [41 (18–65) years vs 29 (16–66) years; p=0.03]. A positive correlation existed between age and pre-transplant cfDNA levels (p=0.018; r=0.177). Neutrophil and platelet engraftment days, chimerism values, frequency of SOS, mucositis, TMA, infectious morbidity, hemorrhagic cystitis, acute/chronic GvHD, and post-transplant relapse were not significantly different among high- and low-cfDNA groups (p>0.05). The total number of febrile days was lower in patients with high cfDNA levels than in the low-cfDNA group [2 (0–15) days vs 3 (0–18) days; p=0.073].

**Figure 1:**
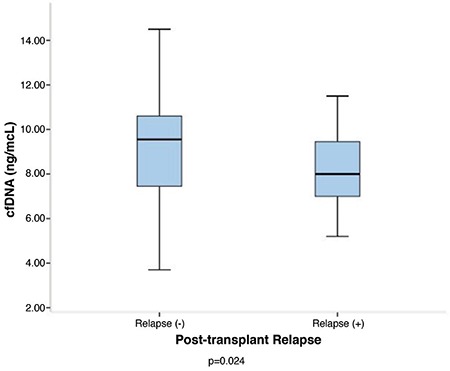
Pre-transplant cfDNA levels were significantly lower in AML patients who experienced post-transplant relapse compared with the patients who stayed in remission (p=0.024). AML: acute myeloid leukemia, cfDNA: cell-free deoxyribonucleic acid

In the subgroup analysis of AML patients, significantly lower pre-transplant cfDNA levels were observed in the course of post-transplant relapse when compared with those who stayed in remission [8 (5.2–11.5) vs 9.6 (3.7–14.5) ng/μL; p=0.024) (Figure 1). No significant association was observed between cfDNA levels and cytogenetical features, including complex karyotype and the mutational status of FLT3, NPM-1, AML1-ETO (RUNX1/RUX1T1), and inv16 (CBFB/MYH11).

**Figure 2:**
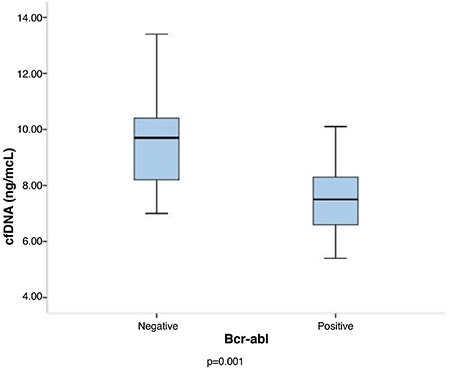
Pre-transplant cfDNA levels were significantly lower in bcr-abl(+) ALL patients compared with bcr-abl(-) patients (p=0.001). ALL: acute lymphoblastic leukemia

In the acute lymphoblastic leukemia (ALL) group, bcr-abl (+) patients had significantly lower pre-transplant cfDNA levels than bcr-abl (-) patients [7.5 (5.4–12.1) vs 9.7 (7–13.4) ng/μL; p=0.001] (Figure 2). Similarly, cfDNA levels were lower in patients who presented abnormal cytogenetics compared with patients with normal karyotype [7.9 (5.4–12.5) vs 10.1 (7–13.4) ng/μL; p=0.038]. SOS was observed more frequently in the low-cfDNA group (p=0.019).

**Figure 3:**
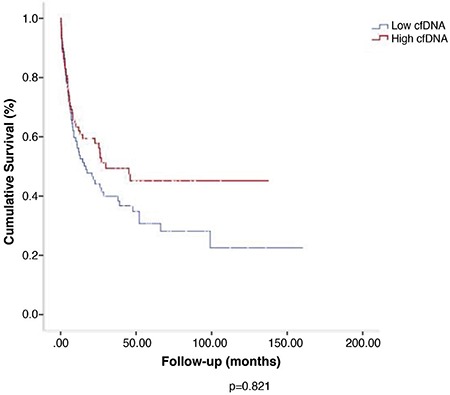
Overall survival was not statistically different between high- and low-cfDNA groups (45.2% vs 22.5%; p=0.821). cfDNA: cell-free deoxyribonucleic acid

Overall survival (OS) was 15.3% in the whole population at the the end of 14.6 (0.2–160.9) months of follow-up. OS was not statistically different between high- and low-cfDNA groups (45.2% vs 22.5%; p=0.821) (Figure 3).

Univariate Cox regression analysis showed that primary diagnosis (p=0.042), pre-transplant disease status (p=0.004), SOS (p<0.001), acute GvHD (p=0.001), chronic GvHD (p<0.001), and relapse (p<0.001) have a significant impact on OS. The significance was confirmed for primary diagnosis (p=0.042), SOS (p<0.001), acute GvHD (p=0.001), chronic GvHD (p=0.009), and post-transplant relapse (p=0.003) in multivariate Cox regression analysis. Pre-transplant cfDNA levels did not present any significant impact on OS (p>0.05).

## DISCUSSION

In this study, the prognostic role of pre-transplant cfDNA levels was investigated in allo-HSCT recipients with acute leukemia. A significant association was identified between cfDNA and age. Pre-transplant cfDNA levels were lower in bcr-abl (+) patients, indicating an adverse correlation between cfDNA and bcr-abl levels. ALL patients with bcr-abl positivity or abnormal cytogenetics presented significantly lower pre-transplant cfDNA levels. Cell-free DNA levels were lower in patients with SOS. In terms of long-term complications, AML patients who presented post-transplant relapse had significantly lower pre-transplant cfDNA levels. OS was not statistically different between high- and low-cfDNA groups. In general, lower serum levels of pre-transplant cfDNA seem to be associated with transplant-related morbidities and may be considered an adverse predictor of prognosis in allo-HSCT recipients.

The amount of cfDNA ranges between 10 to 100 ng/mL in healthy individuals and may be influenced by age, gender, food intake, and diurnal variations. It can circulate as a single molecule or in a complex with cellular or non-cellular components to act as a signaling molecule. Aside from several physiological states such as pregnancy and physical activity, pathological conditions, including inflammation and autoimmune diseases, may present with elevated cfDNA levels. In cancer, cfDNA levels may be increased up to 1000 ng/mL, which draws attention to an association between cfDNA levels and tumor burden (1,4,8,10,11,12,13). A previously reported association between age and cfDNA was confirmed in our study. Teo et al. showed a remarkable consistency between nucleosome signals of cfDNA and redistribution of heterochromatin, which has a role in cellular senescence and aging. In this perspective, cfDNA may be designated as a biomarker of age or a predictor of health status (14).

Circulating tumor DNA analysis requires distinctive attention due to its molecular properties, including low concentration and high fragmentation rate, as well as contamination potential with genomic DNA. The methodologies of cfDNA-based analysis need to be improved because the appropriate source of cfDNA and technical details of sample processing have not been clarified (9). Serum levels of cfDNA are 2–24 times more than plasma levels as a result of the contaminaton of genomic DNA, which is considered a product of WBC lysis during serum processing. As serum cfDNA levels are expected to be more variable than plasma levels, higher serum cfDNA levels are suggested to possibly represent an indirect tumor-related process, whereas plasma levels may be a better indicator of in vivo circulating DNA (4,5,6,9,15,16). Alongside WBC count, several factors may affect cfDNA analysis, such as individualized variations, diurnal rhythm, inflammatory responses, and physical stimuli (1). Although sample preference would be considered an eventual limitation for the present study, we did not mean to discriminate the impact of WBCs on the entire process, considering the potential role of leukocytes in the leukemia microenvironment. From a global perspective, cfDNA may not only be a tumoral or genetic predictor, but also an indicator of inflammation and cytokine stimulation, which is well known to be closely associated with leukocyte activation. The diversity in the methodologies of cfDNA analysis, compared with polymerase chain reaction or next-generation sequencing, may be considered a possible explanation for the contradictory results with the previous studies (5,17,18).

Serum cfDNA levels were incompatible with plasma levels in the previous reports. In a study by Thijssen et al. (15) on patients with colorectal cancer, serum cfDNA was significantly associated with liver metastasis, whereas plasma cfDNA was predictive for recurrence. In concordance with our results, cfDNA levels were decreased in the sera of rheumatoid arthritis (RA) patients. By contrast, several studies stated elevated serum or plasma cfDNA levels in RA patients. Aside from the physiopathological diversity of underlying autoimmune disorders and distinct stages of disease activity at the time of sampling, the role of the heterogeneous methods that were used in sample collection/processing, cfDNA extraction, and quantification should be emphasized. This point of view, which is mainly based on the lack of uniformity and standardization of cfDNA analysis, may lead to a distinct perspective that may particularly elucidate the predictor impact of lower cfDNA levels in the present study (11,12,13).

Several studies underlined the predictor role of cfDNA in endotheliopathies, which shows a significant association of cfDNA with endothelial markers, including syndecan-1 and thrombomodulin (19,20). Endothelial dysfunction, which is indicated to be the major underlying physiopathological mechanism of SOS, may be a reasonable explanation for the association of SOS and pre-transplant cfDNA levels in the current study. However, the predictive role of lower cfDNA levels and its negative correlation with SOS require further evaluation. Sample preference and insufficient number of patients with SOS may partially help elucidate the contradictory results. Simultaneous measures of cfDNA may be more informative for the clarification of the predictive role of cfDNA in the course of SOS.

Several infectious and inflammatory conditions may present with elevated cfDNA levels, which were shown to be correlated with disease severity and mortality (21,22). In addition, cfDNA was associated with the degree of inflammation in viral infections (23). In consideration with these preliminary data, the insignificant association of cfDNA with CMV reactivation may require further verification.

Cell-free DNA levels were elevated in various hematological disorders. High levels of cfDNA and DNA integrity index represented a lower probability of progression-free survival in diffuse large B cell lymphoma patients (8). Cell-free DNA levels were increased in lymphoma patients and correlated with advanced age and stage, poor prognosis, B symptoms, and lactate dehydrogenase levels (4,6,24). The amount of cfDNA was also higher in myeloma patients; this higher amount was shown to be associated with progressive disease (4,25,26).

Several studies reported high cfDNA levels in acute leukemia patients. Cytogenetic and molecular abnormalities such as FLT3 and NPM-1 were identified in cfDNA, suggesting a feasible role for cfDNA in predicting AML prognosis and treatment indications (4). Gao et al. (27) found that not only cfDNA concentrations but also DNA integrity index were elevated in patients with acute leukemia compared with healthy individuals. Given that the DNA integrity index was correlated with disease status, its clinical utility for MRD assessment may also be considered (27). We did not observe any significant association of cfDNA with AML mutational status. However, the adverse correlation of cfDNA with bcr-abl levels and abnormal cytogenetics in ALL patients should be confirmed with large and prospective studies.

Chimerism analysis may be performed in cfDNA in allo-HSCT recipients. Furthermore, because chimerism analysis of cfDNA was shown to predict early relapse in patients with hematological malignancies, monitoring allo-HSCT survivors with cfDNA could be feasible in predicting early relapse (28,29). Lymphoma patients were shown to have detectable cfDNA prior to progression after allo-HSCT (30). The impact of lower cfDNA levels on post-transplant relapse in AML patients and its statistically insignificant impact on OS, which may be associated with inadequate number of patients, require further verification.

In conclusion, cfDNA, which can be considered a non-invasive and repeatable analysis of genetic profiles, may serve a potential tool for diagnosis and prognosis assessment (2,7). Nevertheless, several issues remain to be solved, including standardization of sample collection, processing, and lack of comparability due to inconsistent thresholds (7). We need further understanding of the biological features of cfDNA to determine its true physiological role.

## Figures and Tables

**Table 1:**
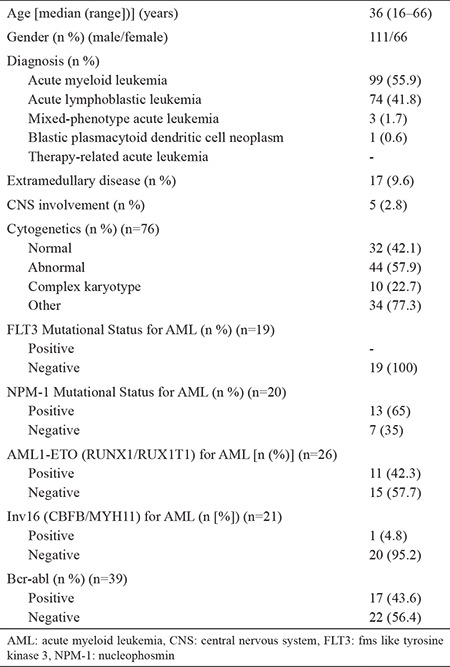
Patient and disease characteristics

**Table 2:**
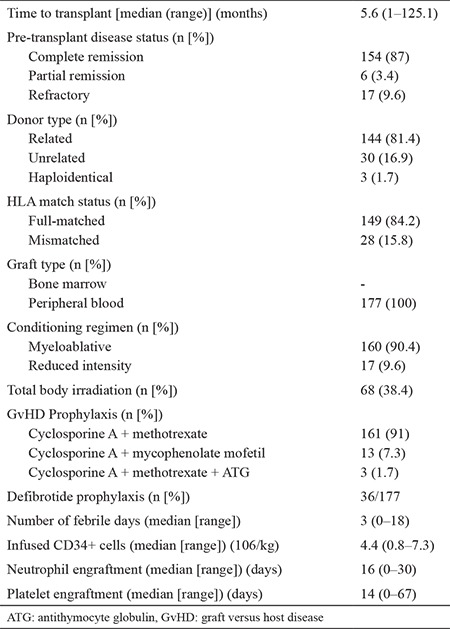
Transplant characteristics

**Table 3:**
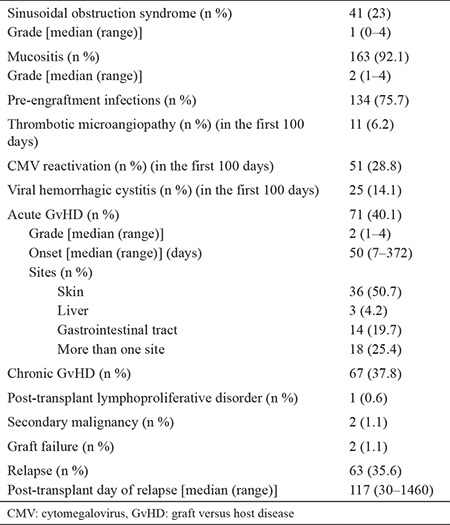
Early and late complications
